# Head and neck cancer therapeutic vaccines: a review

**DOI:** 10.3389/fonc.2026.1773594

**Published:** 2026-05-08

**Authors:** Abigail Huetteman, Guilherme Rabinowits

**Affiliations:** 1 Morsani College of Medicine, University of South Florida, Tampa, FL, United States; 2 Head and Neck – Endocrine Department, Moffitt Cancer Center, Tampa, FL, United States

**Keywords:** cancer immunotherapy, cancer vaccine combination, head and neck squamous cell carcinoma, personalized cancer vaccines, therapeutic vaccines

## Abstract

Head and neck squamous cell carcinoma (HNSCC) accounts for nearly one million new cases of cancer and approximately one half million deaths worldwide each year. The complexity of the tumor microenvironment, the lack of actionable target mutations as well as the increasing resistance of HNSCC to innate immunity pose challenges for development of effective treatment. Personalized cancer vaccines are emerging as a promising treatment strategy in HNSCC. Combining immune checkpoint inhibitor therapy with therapeutic cancer vaccines can enhance immune activation through stimulation of both innate and adaptive immune responses. The ability of therapeutic vaccines to illicit a robust immune response remains promising, however achieving consistent and durable clinical outcome remains a key challenge. In this article, we review the current and emerging strategies in therapeutic vaccination, with a particular focus on personalized cancer vaccines in the treatment of HNSCC.

## 1 Introduction

Cancers occurring in the head and neck differ in both location and variety. Typically, head and neck squamous cell carcinoma (HNSCC) is diagnosed in older patients, and there is a heavy association with tobacco and alcohol use. Additionally, there has been increasing prevalence in younger people associated with Human papilloma virus (HPV) infection (1). HNSCC accounts for nearly one million new cases of cancer and approximately one half million deaths worldwide each year (2). The diagnosis is made through evaluation of physical exam, radiographic evidence and biopsy (1). Both treatment and prognosis depend upon the pathology, biology, and location of the tumor. HPV positivity has been shown to correlate with a better prognosis, including improved response to treatment and overall survival (OS) (1, 3).

Treatment of HNSCC involves surgery, radiation therapy and systemic therapy, with the latter typically recommended for those with locally advanced or metastatic disease. Testing for programmed death-ligand 1 (PD-L1) is now considered to guide systemic therapy options (4).

HNSCC characteristically exhibits heterogeneity among its molecular characteristics, cellular phenotypes, and complexity of the tumor microenvironment (TME). Given the intricate and rapidly changing genomic nature of tumor biology in HNSCC, there are limited concrete or feasible targets for therapy. Prior attempts to target mutations such as Phosphatidylinositol-4,5-bisphosphate 3-kinase catalytic subunit alpha (PIK3CA) or Epidermal growth factor receptor (EGFR) have been of limited activity (5, 6). The development of immune checkpoint inhibitor (ICI) therapy introduced an alteration in the conventional treatment framework and is now considered standard of care for locally advanced HNSCC patients undergoing surgery and for those with recurrent or metastatic disease (4, 7).

The use of ICIs as a treatment modality in HNSCC has provided evidence for pre-existing immunity against autologous tumor cells. In genomic analysis of HNSCC, no widely shared oncogenic driver mutations have been identified, but a high tumor mutation burden as well as high infiltration of immune cells into the TME are common occurrences (5, 8, 9). Together, these highlight the rationale of ICI in the management of HNSCC. However, only a small number of patients respond to ICI monotherapy (7, 10). In recent years, therapeutic vaccines have emerged as another promising candidate for treatment of HNSCC and various strategies have been explored. Most commonly, both tumor-associated antigens (TAAs) such as EGFR, Melanoma antigen encoding gene (MAGE), p53, p16, and Mucin (MUC) 1, and tumor-specific antigens (TSAs) such as HPV E6 and E7 and Epstein-Barr virus (EBV) latent membrane protein (LMP)-2 (11), have been the object of these approaches.

Ongoing research is exploring the combination of therapeutic vaccines and ICIs as cancer therapy to overcome their limited activity as monotherapy. A major challenge of the ICIs is their inability to directly infiltrate tumor with immune cells and overcome the immunosuppressive TME. In contrast, while cancer vaccines can produce robust tumor immune cell infiltration, targeting specific tumor antigens, it is often accompanied by the upregulation of immune checkpoint molecules like PD-L1 and cytotoxic T-lymphocyte associated protein 4 (CTLA-4) resulting in T cell dysfunction, making them less able to overcome immune regulatory mechanisms. Taken together, cancer vaccines can increase infiltration of immune cells into the TME while ICIs can prevent and reverse dysfunction of immune cells through blockade of PD-L1 and CTLA-4 (12) supporting the rationale for the combination.

As research progresses, it is becoming increasingly clear that, in addition to HNSCC’s ability to undergo “immune evasion” through which tumor cells bypass host immunosurveillance, each patient’s tumor possesses a unique mutational scheme. Together, this underscores the importance of a more personalized, tumor-specific approach to therapeutic vaccination in conjunction with ICI therapy.

In the current literature, several reviews have addressed individual aspects of therapeutic vaccination, however comprehensive synthesis is lacking. Historically, most therapeutic cancer vaccine strategies have shown limited clinical benefit, and personalized cancer vaccines have emerged as a compelling alternative, capable of overcoming key limitations of tumor-associated and other tumor-specific antigen–based approaches. As results from ongoing clinical trials emerge, a comprehensive review is needed to integrate new evidence with current knowledge, highlight the rising potential of personalized vaccines, discuss vaccination strategies currently in development, and address major challenges that must be overcome to support their progression toward its use in clinical practice.

## 2 Principles of therapeutic vaccines in HNSCC

To gain deeper insight into how immunotherapy can serve as a treatment modality in HNSCC, a clear understanding of how the pathophysiology of immunity becomes disordered in these tumors is essential.

Our immune system can recognize and respond against cancer cells in two distinct ways: through TAAs or TSAs. TAAs are normal proteins that are abnormally expressed in cancer cells but are also found in healthy tissue often at lower levels. They are “self-antigens,” therefore are not highly immunogenic as any T cells capable of eliciting a robust immune response against them are eliminated through negative selection. Using TAAs as a therapeutic target additionally carries a risk of autoimmune toxicity (13, 14). TSAs on the other hand, are expressed exclusively by cancer cells and are recognized as “non-self” by the immune system. There are different subsets of TSAs, including viral antigens in viral-associated cancers (such as HPV E6 and E7), and neoantigens (13, 14).

The immune system processes both TAAs and TSAs in identical ways. First, they are broken down into short peptides through proteasome degradation and loaded onto major histocompatibility complex (MHC) class I molecules. These peptide-MHC complexes are displayed on cell surface for recognition by CD8+ T cells. Both dendritic cell uptake and cross presentation as well as direct presentation of antigens on tumor cell surface are essential for antigen recognition and T cell activation (14–17). These reactivated T lymphocytes then carry out cytotoxic processes, inducing death of tumor cells, a process known as cell-mediated toxicity, which has been the core method of exploration and development of an anti-tumor mechanism in therapeutic vaccines (15).

A subset of TSAs, called neoantigens are formed and processed in a similar way. During the process of neoplasm formation, short peptide sequences generated through errors in transcription, post-translation modification and other genomic abnormalities, called neoantigens, are subsequently released into circulation (15, 17). Because neoantigens are recognized as “non-self” by the immune system, the newly mutated segments may acquire the ability to bind to MHC complexes. Subsequently, their presentation to the host T cells leads to T cell activation and differentiation into memory T cells and effector T cells (15, 18). Meanwhile, CD4+ naïve T cells are activated by T-cell receptor recognition of MHC-II of dendritic cells. Secretion of cytokines such as interferon (IFN)- y and IL-12 help differentiate CD4+ naive T cells into Th1 cells, which also assists CD8+ cells to differentiate into memory and effector T cells (9). Cancer vaccine therapy utilizes this increase in CD4+ and CD8+ T cells to eradicate tumor cells through the recognition of neoantigens.

A subset of neoantigens, called neojunctions are formed from aberrant RNA splicing rather than DNA mutations. Whereas “canonical” antigens are those that are mutation-derived, “non-canonical” antigens can be formed from gene fusions, frameshifts, and splice variants among other alterations (19). Neojunctions typically arise from shared variations in splicing machinery, meaning the increased commonality of these mutations across patients enables broadly applicable therapeutic strategies, providing an alternative for lengthy, individualized therapeutic approaches and are a growing area of exploration in therapeutic cancer vaccine development (19–21).

Despite the existence of naturally occurring immune reactions against neoantigens, there is still increasing resistance of HNSCC to anti-tumor immune responses. Tumors have demonstrated the ability to undergo “immune evasion” which occurs through several mechanisms: first, the upregulation of immune checkpoint molecules such as PD-L1 induces T cell exhaustion and impaired cytotoxic T cell response (22). Second, tumors demonstrate infiltration and recruitment of several immunosuppressive cells including cancer-associated fibroblasts, tumor-associated neutrophils, tumor-associated macrophages (TAMs), myeloid-derived suppressor cells (MDSCs), and regulatory T cells (Tregs) (5, 15). This infiltration causes secretion of immunosuppressive cytokines, including transforming growth factor-beta (TGF-beta), prostaglandin E2 (PGE2) and interleukin-10 (IL-10), and inhibition of effector T cell function (22, 23). Of note, there is conflicting data in regard to Tregs cell infiltration and its prognostic association, with some studies reporting an improved outcome, suggesting that Tregs may reflect broader immune activation rather than direct immune suppression in certain situations (24, 25). Third, the effective impairment of antigen presentation through the downregulation of MHC-1 leads to reduced cancer immunogenicity through reduced recognition by cytotoxic T cells and establishment of an “cold” immunosuppressive TME (26, 27). As tumor cells become less effectively recognized by the immune system, they facilitate immunotherapy resistance as well as progression of HNSCC. In the absence of a functioning immune pathway, several vaccine strategies have become ineffective (15).

## 3 HPV-targeting cancer vaccine

HPV-positive tumors arise from transcriptionally active HPV infections. The virus promotes carcinogenesis through the expression of E6 and E7 oncoproteins, which inactivate the tumor suppressor proteins p53 and retinoblastoma (Rb), respectively, leading to disruption in cell cycle regulation and resulting in uncontrolled cell proliferation. Unlike prophylactic HPV vaccines that aim to prevent HPV infection, targeting L1 capsid protein of the HPV, therapeutic HPV vaccines target high-risk HPV oncoproteins E6 and E7, which are consistently expressed in infected cells and are involved in development of cancer (28, 29). Several vaccines targeting HPV-positive HNSCC have been in development and have shown promising data in early-stage clinical trials (**Table 1**):

**Table 1:** Therapeutic vaccine platforms in HNSCC.

Vaccine name	Vaccine type	Antigen target(s)	Clinical setting	Phase (trial ID)	Combination strategy	Key clinical outcomes	Safety profile
ISA101b (33) (NCT02426892) N=24 (22 OPC)	Synthetic long peptide vaccine	HPV E6/E7 oncoproteins	Recurrent/Metastatic HPV+HNSCC	Phase II	Anti-PD-1	ORR 33%; median PFS 2.66 months; median OS 15.3 months	Well tolerated; limited grade ≥3 toxicity
MEDI0457 (INO-3112) (NCT03162224) (36) N=29	DNA plasmid vaccine + electroporation	HPV E6/E7 oncoproteins	Locally advanced; Recurrent/Metastatic HPV+ HNSCC	Phase I/II	Anti-PD-L1	ORR 27.6%; median PFS 3.5 months; median OS 29.2 months	Mostly grade 1–2 TRAEs
Lenti-HPV-07 (NCT06319963) (37) N=72 (36 OPC)	Lentiviral vector vaccine	HPV E6/E7 oncoproteins	Locally advanced & metastatic HPV+ HNSCC and cervical cancers	Phase I/II (ongoing)	Monotherapy	Results pending	Safety data pending
VB10.16 (38) (NCT02529930)	DNA vaccine with APC-targeting delivery	HPV E6/E7 oncoproteins	HPV16-driven malignancies (platform expansion to HNSCC)	Phase I/II	Anti PD-L1	Proof-of-mechanism efficacy in cervical cancer; HNSCC data pending	Favorable tolerability in early studies
PDS0101 (NCT04260126) (40) N=53	Liposomal vaccine	HPV E6/E7 oncoproteins	Recurrent/Metastatic HPV+ HNSCC	Phase II	Anti-PD-1	ORR 35.8%; median PFS 6.3 months; median OS 30 months	Well tolerated with limited grade ≥3 toxicity
CUE-101 (NCT03978689) (41, 42) N=80	Modular T cell engager - fusion protein of HLA, peptide epitope and attenuated IL2 vaccine	HPV E7 oncoproteins	Recurrent/Metastatic HPV+ HNSCC	Phase I	+/- anti-PD-1	Monotherapy: ORR 5%; median OS 20.8 months. Combination: ORR 47%; median PFS 5.8 months; median OS not reached	Well tolerated with limited grade ≥3 toxicity
BNT113 (44) (NCT04534205) N=15	Uridine-modified mRNA lipoplex vaccine	HPV E6/E7 oncoproteins	Recurrent/Metastatic HPV+ HNSCC	Phase II	Anit-PD-1+/-BNT113	ORR 40%; median PFS 3.9 months; median OS 22.6 months	Well tolerated with limited grade ≥3 toxicity
MVA-EL (52) (NCT01147991) N=16	Modified vaccinia Ankara viral vector	EBNA1–LMP2	EBV+ malignancies	Phase I	Monotherapy	Demonstrated immunogenicity; limited clinical efficacy signal	Safe; mild vaccine-related AEs
Allovax (CRCL + AlloStim) (NCT01998542) (58) N=10	Chaperone-rich cell lysate + allogeneic Th1-like cells	Autologous tumor antigens (broad repertoire)	Heavily pretreated R/M HNSCC	Phase II	Monotherapy	~50% with tumor reduction in early cohort; increased CD3+ infiltration	Generally tolerable in early reports
GEN-009 (60) (NCT03633110) N=15	Adjuvanted personalized neoantigen peptide vaccine (ATLAS-selected)	Up to 20 patient-specific neoantigens	Post-curative intent treatment; advanced solid tumors	Phase I	Anti-PD-1	Broad neoantigen-specific T-cell responses; epitope spreading observed	Minimal additive toxicity
MVX-ONCO-1 (62) (NCT02193503)	Autologous irradiated tumor cells + GM-CSF capsules	Autologous tumor antigens	Advanced refractory solid tumors including R/M HNSCC	Phase I	Monotherapy	PR/SD observed; prolonged survival in select HNSCC cases	Generally well tolerated
TG4050 (63, 64) (NCT04183166) N=33	Modified vaccinia Ankara viral vector	Up to 30 patient-specific neoantigens	Adjuvant vs at recurrence HNSCC)	Randomized Phase I	Monotherapy (adjuvant arm)	100% DFS in adjuvant arm at ~28 months; robust polyepitopic T-cell priming	Grade 1–2 AEs; injection site reactions most common
mRNA-4157 (69, 70) (NCT03313778) N=50 (cohort C: R/M HPV- HNSCC)	Lipid nanoparticle encapsulated mRNA vaccine	Up to 34 patient-specific neoantigens	Advanced solid tumors, including HPV- HNSCC	Phase I	adjuvant (monotherapy) R/M disease: + anti-PD-1	R/M HNSCC: ORR 27/3%; median PFS 4 months; median OS 26 months	No dose-limiting toxicities reported
PNeoVCA (66) (NCT05269381)	Personalized neoantigen peptide vaccine	Up to 20 patient-specific peptides	Advanced solid tumors	Phase I (ongoing)	Anti-PD-1	Feasibility demonstrated; efficacy data pending	No major safety signals reported
YE-NEO-001 (QUILT-2.025) (NCT03552718)	Personalized yeast-based neoepitope vaccine	Multiple patient-specific neoepitopes	Post-curative therapy surveillance/MRD	Phase I (ongoing)	Monotherapy	Results pending	Safety data pending
VB10.NEO (67) (NCT05018273) N=26	DNA-based personalized neoantigen vaccine	Frameshift + SNV-derived neoantigens	Advanced solid tumors	Phase Ib	Anti-PD-L1	SD ~34.8%; neoantigen-specific T-cell expansion in responders	Mostly grade 1–2 AEs; no serious TRAEs
PGV001 (68) (NCT02721043) N=14	PCV through use of computational pipeline OpenVax	Up to 10 patient-specific neoantigens	Adjuvant setting for patients with high risk solid and hematologic malignancies	Pilot	Monotherapy	Feasibility and safety demonstrated with 100% of vaccinated patients developing targeted B and T cell responses	Only grade 1–2 TRAEs reported.

HNSCC, head and neck squamous cell carcinoma; HPV, human papillomavirus; EBV, Epstein–Barr virus; LA, locally advanced; R/M, recurrent/metastatic; MRD, minimal residual disease; ORR, overall response rate; PFS, progression-free survival; OS, overall survival; SD, stable disease; PR, partial response; TRAE, treatment-related adverse event.

- ISA 101b, a synthetic long peptide (SLP)-based vaccine derived from E6 and E7, contains 13 overlapping SLPs (nine from E6 and four from E7) which taken together cover the complete sequence of E6 and E7. When delivered to APCs, robust HPV-specific T-cell responses can be elicited (30). A landmark trial exploring ISA 101b in high-grade vulvar intraepithelial neoplasia demonstrated clinical activity (31), potentially resulting from a virus-specific T-cell immune response. In a phase II trial, ISA 101b combined with the anti-PD-1 antibody cemiplimab for recurrent/metastatic HPV16+ oropharyngeal cancer achieved an overall response rate (ORR) of 11.5% in patients who had previously failed ICI monotherapy (32). Another study evaluating ISA 101b with nivolumab across different types of recurrent HPV-positive cancers (24 total patients, of which 22 had oropharyngeal cancers) demonstrated an ORR of 33% and OS of 17.5 months, which successfully met the study’s primary endpoint. Additionally, grade 3–4 toxicities were reported in only two patients (30). Updated results from the long-term follow-up of this study showed a median duration of response of 11.2 months, a median OS of 15.3 months and a 2-year OS of 33%, highlighting the potential clinical benefit of combining ISA 101b with an ICI (33). The observed clinical responses, in addition to enhanced T-cell immunity purports the idea that adaptive anti-tumor immunity can be enhanced through expansion of HPV-specific T-cells by ISA 101b and preservation of T-cell function by anti-PD-1 therapy (34).
- MEDI0457 (INO-3112) is a DNA based therapeutic vaccine with two components: VGX-3100, a synthetic DNA plasmid targeting HPV16–18 E6 and E7 through SynCon optimization to generate DNA sequences, and INO-9012, a DNA plasmid containing recombinant interleukin (IL)-12. This combination is delivered by electroporation with the CELLECTRA device, a constant current device that delivers three precise electrical pulses at the injection site resulting in improved gene delivery and increased antigen expression, which in turn elicits a stronger immune response. This treatment modality was evaluated in a phase I/II study with patients with HPV-positive locally advanced HNSCC who received administration of this experimental agent either in the perioperative setting (cohort I) or after chemotherapy and radiation therapy (cohort II). Results of this trial showed an advantageous safety profile with no serious treatment-related adverse events (TRAEs). Additionally, an anti-HPV humoral response was observed in all patients in both cohorts and follow-up revealed persistence of the response for up to 23 months (35). Another phase Ib/IIa study investigated MEDI0457 in conjunction with the PD-L1 antibody durvalumab in relapsed/metastatic (R/M) HNSCC patients. The ORR among the 29 evaluable patients was 27.6% (four complete and four partial responses), with responses independent of PD-L1 expression. The median progression free survival (PFS) was 3.5 months, the 16-week disease control rate was 44.8%, and the median OS was 29.2 months. TRAEs occurred in 80% of the patients, mostly grades 1 and 2. An increase in CD8+ T cells HPV16/18-specific was also observed (36).
- Lenti-HPV-07 is a therapeutic vaccine targeting HPV-induced cancers based on the lentiviral vector technology developed by Pasteur-TheraVectys Joint Laboratory. Preclinical studies have demonstrated its ability to induce a robust cellular immune response against HPV16 and HPV18 antigens (37). A phase I/IIa clinical trial evaluating the efficacy and safety of Lenti-HPV-07 in patients with HPV-related locally advanced and metastatic cervical and oropharyngeal cancers is currently ongoing, and results are eagerly awaited (NCT06319963).
- VB10.16 is a HPV E6/E7 targeted therapy that utilizes a CCL3L1-targeted antigen delivery to APCs. Phase I/IIa trials explored its use in high-grade/advanced HPV16-positive cervical cancer in combination with atezolizumab. No serious adverse events were reported. Forty seven percent of patients demonstrated HPV clearance, 94% showed reduction in lesion size, and 59% showed regression into a lower-grade lesion. There was a statistically significant association between interferon (IFN)y T-cell responses and reduction in lesion size (38).
- PDS0101 is a liposomal therapeutic vaccine composed of the enantiospecific cationic lipid nanoparticle R-DOTAP (Versamune) and HLA-unrestricted HPV16 E6/E7 peptide antigens. It is designed to generate potent CD4+and CD8+ T-cell responses against HPV-associated malignancies. In a phase 1/2 trial (NCT04287868), PDS0101 was evaluated in combination with PDS01ADC and bintrafusp alfa in patients with advanced HPV-associated cancers including oropharyngeal SCC. The combination demonstrated an ORR of 62.5%. Grade 3–4 treatment-related adverse events occurred in 52% of patients (39). In the phase 2 trial VERSATILE-002, PDS0101 was evaluated in combination with pembrolizumab in HPV16-positive R/M HNSCC in ICI-naïve and ICI-refractory patients. The median PFS observed was 10.4 months (historically compared to 2–3 months in populations treated with ICI monotherapy), and the estimated OS at 12 months was 87.1%. This therapy was well tolerated with no grade IV-V adverse events and only 6.3% grade III events (40).
- CUE-101 is an emerging therapeutic fusion vaccine composed of a HLA-A*0201, a peptide epitope derived from the HPV16 E7 protein and 4 molecules of attenuated human IL-2, developed as an off-the-shelf therapy for HPV16-positive malignancies designed to selectively activate and expand HPV-specific CD8+ T-cells (41). A phase 1 clinical trial (NCT03978689) evaluated the safety and tolerability of CUE-101 in HPV16 positive R/M HNSCC patients, administered as monotherapy or in combination with pembrolizumab. Among the 19 evaluable patients treated with the recommended phase 2 dose of CUE-101 with pembrolizumab, the ORR was 47%, disease control rate (DCR) was 74% and mPFS was 5.8 months. The median OS was not reached for the combination arm, and it was 20.8 months for the CUE-101 monotherapy arm (42).
- BNT113, a therapeutic mRNA lipoplex (RNA-LPX) vaccine encoding HPV16 E6/E7 proteins designed to stimulate antigen-specific T cell responses against HPV-associated cancers. The RNA-LPX platform works through utilization of positively charged cationic lipids in conjunction with negatively charged mRNA that facilitates uptake by APCs. This then targets dendritic cells that promote robust CD4+ and CD8+ T-cell responses against the E6/E7 antigens (43). Preliminary results of a phase 2/3 clinical trial AHEAD-MERIT (NCT 04534205) evaluating pembrolizumab +/- BNT113 demonstrated unconfirmed partial response of 40%, median PFS of 3.9 months and median OS 22.6 months among the safety run in 15 patients receiving the treatment combination. Treatment was well tolerated with limited grade ≥3 and no treatment-related deaths. Two-thirds of the patients showed *de novo* vaccine-induced T-cell responses against E6 and E7 oncoproteins, with HPV16 E7-specific CD8+ T cells reaching 5.2% of peripheral CD8+ T cells after 10 months in a patient with prolonged stable disease. The study is ongoing, and results are eagerly awaited (44).

While E6 and E7 make ideal targetable tumor antigens, and studies have shown the induction of HPV-specific immune responses in association with therapeutic vaccination, their role in preventing tumor progression and benefitting long-term survival remains limited. This may be attributable to several factors. First, tumor progression is known to exacerbate immune dysfunction. Studies of recurrent HNSCC have reported significant depletion of stem-like TCF1+ CD8+ T cells, central drivers of HPV-specific immunity that sustains T-cell responses under chronic antigen exposure and differentiation into HPV-specific effector-like T-cells (45). Additionally, in patients with recurrent disease, HPV specific T cells frequently exhibit phenotypic markers of exhaustion (46) and can display diminished E6 and E7 specific T-cell responses (47). Heterogeneity within the tumor further contributes to immune resistance, in part through producing variable patterns of antigen expression. Notably, HPV-specific T cells can respond against a wide array of HPV antigens beyond E6 and E7 (48). Together, these highlight multiple barriers that likely contribute to the limited efficacy observed from HPV-targeting therapies.

## 4 Epstein-Barr virus-targeting cancer vaccine

Therapeutic vaccination strategies in nasopharyngeal carcinoma (NPC) leverage the strong association between EBV infection and tumor pathogenesis. Key targets include the Epstein-Barr virus nuclear antigen-1 (EBNA1) and the Epstein Barr virus LMP1 and 2 (49). EBNA1, expressed in all EBV-associated tumors, promotes oncogenesis by disrupting tumor suppression and inducing DNA damage. LMP1/2 are tumor necrosis factor receptors involved in multiple signaling pathways that drive immunogenicity and oncogenesis. Through eliciting robust T cell responses against these antigens, these therapeutic vaccines aim to promote selective killing of EBV-infected tumor cells (50).

A promising candidate for EBV targeted therapeutic vaccination is MVA-EL (**Table 1**), a modified vaccinia virus Ankara with EBNA1-LMP2 fusion protein. The EBNA1-LMP2 fusion protein has been altered to destroy carcinogenic properties of its constituents while retaining ability to generate CD4 and CD8 immune responses (51). A phase 1 trial with 16 patients with EBV-positive malignancy, either in first remission or with persisting disease for which no standard therapy was available, received three intradermal vaccinations of MVA-EL at 3-weekly intervals. The vaccine was proven to be safe and demonstrated immunogenicity across diverse ethnic backgrounds, thereby supporting its further testing against other EBV^+^ cancers beyond NPC (52).

Despite the strong rationale for use of EBV as a target for therapeutic vaccination, it has proven difficult to translate into successful clinical outcomes. Key challenges in successful vaccine development include the biological complexity of EBV, the prolonged period between EBV infection and tumor development, and persistence of latent infection within multiple cell types including B cells, epithelial cells, and NK/T-cells (53). Additionally, EBV employs several immune-evasion strategies including upregulation of PD-L1, suppression of interferon signaling, modulating interleukin secretion, and disruption of MHC antigen presentation, which together impair effective T-cell responses and undermine vaccine efficacy (54). These mechanisms may help explain why clinical efficacy is limited despite the measurable immune response that has been demonstrated in early phase clinical trials.

## 5 Personalized cancer vaccines

Because each tumor exhibits numerous and unique set of peptide segments (neoantigens) that can serve as a target in the development of vaccine therapy, a role has emerged for the use of PCVs. PCVs rely on the specific biology unique to the individual tumor to induce an anti-tumor immune response (11). Neoantigen mutations evolve as the tumor evolves, making each set of mutations unique to the individual cancer (18). It is through the analyses of these peptides presented on MHC molecules that neoantigens are identified as potential targets of PCVs (9).

Several PCVs have been designed and are being tested as a potential treatment modality for HNSCC (**Table 1**). Some are reviewed here:

- Allovax, a PCV being explored in a phase II clinical trial (NCT01998542), combines Chaperone Rich Cell Lysate (CRCL) with AlloStim technology. CRCL is a vaccine preparation that contains several types of molecular chaperones such as heat shock proteins (HSPs) that are extracted from tumor cells. These chaperones then deliver tumor antigens to dendritic cells to activate tumor-specific cytotoxicity (55). It is used in conjunction with AlloStim, which is a living cell therapy that obtains allogeneic immune cells and purifies, expands, and differentiates them into mature Th1-like cells. Together, these create a “host-vs-graft” response, which in turn, stimulates the immune system to create a “host-vs-tumor” response. This facilitates the development of a robust, host-driven, anti-tumor immune response (56, 57). By focusing on the development of immunity specific to the individual tumor, tumor immune tolerance can be overcome and anti-tumor response strengthened in the host. Preliminary published data (NCT01998542) has shown that half of the 10 heavily pretreated, chemotherapy-refractory, R/M HNSCC patients who received the vaccine, developed visible tumor reduction associated with increase in CD3+ immune cell infiltration, and down-regulation of CTLA-4 (58), an immune checkpoint receptor that acts as a negative regulator of T-cell activation that prevents excessive immune response (59).
- GEN-009 is an adjuvanted PCV containing up to 20 neoantigens selected by ATLAS (Antigen Lead Acquisition System), an ex vivo bioassay screening autologous T cells for immune responses against both neoantigens and inhibigens (peptides that accelerate tumor progression through immune suppression) (60). Its use in HNSCC is currently being evaluated in patients who have completed curative-intent therapy (surgery, radiation therapy, and/or neoadjuvant/adjuvant chemotherapy) with no evidence of disease at the time of GEN-009. Preliminary activity of GEN-009 in combination with anti-PD-1 in 15 patients with advanced solid tumors demonstrated induction of broad neoantigen-specific immune responses and epitope spreading with little additive toxicity supporting further study (NCT03633110) (61).
- MVX-ONCO-1 is a PCV that combines irradiated autologous tumor cells and an immune modulator - genetically modified allogeneic cells producing granulocyte-macrophage colony-stimulating factor (GM-CSF) contained within a capsule. Each treatment consists of irradiated tumor cells, which are injected under the skin and two capsules of GM-CSF cells implanted subcutaneously for one week. An open-label, single-arm, first-in-human phase I study with MVX-ONCO-1 in patients with advanced refractory solid cancer showed clinical benefit in most patients, including partial responses, stable disease and prolonged survival. In R/M HNSCC, one patient achieved a partial response, whereas another survived for more than 7 years without anticancer therapy for over 5 years supporting further studies on this patient population. The vaccine was overall well tolerated, with most adverse events observed related to disease progression (62).
- TG4050, a PCV using a Modified Vaccinia Ankara viral vector was tested in surgically resected, stage III or IV, HPV-negative HNSCC patients. A PCV for each patient was manufactured with up to 30 neoantigens identified using a machine learning algorithm from next generation sequencing (NGS) data. Patients randomized to arm A (n=17) received the PCV after completion of primary treatment and those randomized to arm B (n=16) received the PCV in the event of relapse, in conjunction with second line therapy. Tumor location was oral cavity in 24 patients (72.7%), hypopharynx and oropharynx in 4 patients (12.1%), respectively and larynx in one patient (3.0%). TG4050 was found to be safe with only grade 1–2 adverse events observed. The most frequently reported TRAEs were injection site reactions. After a median follow-up of 28.5 months, all patients receiving TG4050 in arm A remained disease-free whereas 3 patients in arm B relapsed. Immune monitoring demonstrated priming of a polyepitopic T cell response against the PCV in 100% of patients in arm A (63, 64). This study suggests that cancer vaccine’s efficacy may be better when treating tumors with low tumor mutational burden or those with minimal residual disease, as patients in arm A of this study received TG4050 as monotherapy. Larger tumor mutational burden is associated with a higher degree of immune suppression, requiring combination therapy (63–65).
- PNeoVCA, a PCV composed of 20 individual peptides, is being studied as part of an ongoing phase I single arm clinical trial to assess the safety, feasibility, and immunogenicity of PCVs in combination with pembrolizumab in patients with advanced solid cancers (NCT05269381). This vaccine was developed using REAL-neo, an informatics workflow that identifies more potent neoantigen candidates on the patient’s own tumor DNA and mRNA sequencing data. The first cohort received the first dose level of PNeoVCA without any reported toxicity (66).
- QUILT-2.025 is a phase 1 clinical trial evaluating the safety, the recommended phase 2 dose, and the preliminary efficacy of a personalized neoepitope yeast-based vaccine (YE-NEO-001) in subjects who have completed potentially curative therapy for their solid cancer and who would otherwise be entering a period of surveillance for recurrent disease (NCT03552718). YE-NEO-001 was engineered to express multiple neoepitopes based on an individual subject’s tumor molecular profile. Results of this trial are eagerly awaited.
- VB10.NEO is a DNA based PCV developed through the NeoSELECT platform, which analyzes RNA and circulating tumor DNA and incorporates both frameshift and single nucleotide variants into its neoantigens. Like VB10.16 for HPV-positive cancers, VB10.NEO utilizes a similar rationale through the use of CCL3L1-targeted antigen delivery to APCs. A phase 1B dose escalation clinical trial tested VB10.NEO at varying doses in combination with the anti-PD-L1 atezolizumab in 26 heavily pre-treated patients with advanced solid tumors. Across all dose levels, VB10.NEO induced robust and durable neoantigen-specific immune responses. T cell receptor sequencing showed persistent T cell clone expansion in 9 of 11 patients, indicating durable immune responses. All patients achieving stable disease (34.8%) exhibited neoantigen-specific immune responses. Additionally, VB10.NEO was well tolerated with mainly grade 1–2 adverse events reported. No serious TRAEs or deaths occurred (67).
- PGV001 is a PCV developed through use of computational pipeline OpenVax and designed to target up to 10 patient-specific neoantigens. It was studied in the adjuvant setting in patients with a variety of solid and hematologic malignancies, including HNSCC, who had previously received curative intent therapy and were estimated to have a greater than 30% risk of recurrence within 5 years. This study found that 100% of patients generated antigen-specific T-cell and B-cell responses, which highlights the promise of the OpenVax system for accurate neoantigen prediction (68).
- mRNA-4157 is a PCV using lipid nanoparticle-encapsulated mRNA that encodes up to 34 neoantigens. In a phase I trial for patients with various advanced solid tumors, including HPV negative HNSCC, mRNA-4157 showed preliminary efficacy as monotherapy in the adjuvant and in combination with pembrolizumab in the R/M settings. Among the 22 patients with ICI-naïve R/M HNSCC, the ORR with the combination was 27.3%, the DCR was 53.6% and the median PFS and OS were 4 months and 26 months, respectively, with a favorable safety profile (14% experiencing G3 TRAEs). These promising results need to be confirmed in a randomized trial (69, 70).

## 6 Challenges emerging for PCV therapy in HNSCC

As previously stated, HNSCC demonstrates a complex and heterogeneous TME that does not possess a commonality of oncogenic driver mutations or consistent mechanism for evading host immunity. Together, these raise potential challenges in both the development of PCVs and their utility in clinical practice.

PCVs targeting neoantigens are designed to boost endogenous or elicit new T cell responses against tumor-specific mutations, thereby raising anticancer immunity to clinically relevant levels and improve/expand the efficacy of ICIs, making “cold” tumors more responsive to therapy (71, 72).

Despite this strong rationale, therapeutic cancer vaccines have demonstrated overall low immunogenicity (73). This is likely due to intrinsic features of the selected neoantigens, including similarity to host peptides, the low antigen load delivered and their suboptimal immunostimulatory capacity (71, 73). It has been previously demonstrated through tumor-specific T cells profiling that patients typically mount an immune response against less than 1% of the entirety of their cancer mutations (17).

Importantly, even when studies have successfully generated robust T cell responses to specific neoantigens, translation into clinical benefit is not consistently observed, supporting the notion that the generation of neoantigen-specific T cell responses alone is not sufficient (74, 75). For example, in a murine colon carcinoma model, a neoantigen derived from a mutation in the *SMC3* gene induced a significant CD8+ T cell response, however, these T cells failed to identify or attack the tumor cells (74). Similarly, in a lung cancer model, CD8+ T cell accumulation within tumors after ICI therapy combined with vaccination was observed, however the response to treatment was insufficient (75). The limited clinical efficacy observed is likely due to tumor-mediated immune resistance mechanisms, including an immunosuppressive TME, T-cell exhaustion, and impaired antigen presentation machinery, as previously discussed. These examples question the utility of tumor antigens as a strategy for the development of efficacious antitumor vaccines that elicit clinically meaningful immune responses.

The identification of neoantigens that illicit a significant immune response remains a significant challenge. The development of NGS has helped in the provision of a wealth of genetic information of the individual tumor and has aided in the prediction of potential peptides to be used as neoantigens, despite a limited portion achieving recognition by the patient’s immune system (73). Whole genome and exome sequencing (WGS and WES, respectively) along with RNA sequencing (RNA-seq) are the most used approaches in neoantigen and HLA-binding prediction. These, in combination with inference of mutated peptides based on somatic mutation data, and prediction of HLA binding and antigen presentation, represent the four components of a majority of neoantigen prediction bioinformatic pipelines (76).

However, these algorithms still struggle to identify neoantigens that are reliably expressed on the tumor cell surface and elicit robust immune responses (77). A major limitation is that predicted HLA-binding affinity has a relatively low positive predictive value. In one study, only 22% of predicted neoantigens demonstrated successful HLA-binding (78). Additionally, high false positive rate for neoantigen prediction further inhibits effective vaccine development (79). This is of significant concern, particularly for patients with limited treatment windows. To address this challenge, one potential strategy involves direct analysis of the HLA ligandome to assist with development of tumor-specific vaccines. A previous study of oropharyngeal carcinoma used analysis of HLA binding sites to build a repository of peptides for future use in development of semi-personalized vaccines, although this study did not assess the immunogenicity of these peptides (80).

Importantly, accurate HLA binding prediction alone is insufficient. Adequate tumor expression of neoantigens through increased RNA translation into protein is required for neoantigens to be properly processed and presented on MHC molecules (81, 82). Even when multiple neoantigens are successfully presented, the immune system does not elicit similar immune responses. Certain neoantigens will trigger stronger T cell responses than others, a process known as immunodominance (83).

Over time, tumor evolution will cause certain neoantigens to no longer be present, and alterations in the TME can affect immunogenicity of neoantigens (84). Identifying which antigens will be expressed and subsequently activate the immune system remains a challenge.

Finally, there is the added logistical undertaking in the process of translating each individual tumor’s mutations into development of a PCV. This increases the challenge of addressing the need for both personalization and readily available therapeutic approaches (17). Each vaccine requires a careful review of the NGS data, the prediction of potential neoepitopes, and the manufacturing of each individual vaccine. Time is of the essence as delays in initiating therapy may negatively impact patients’ outcome. The current methodology used to select potentially targetable epitopes must be improved for efficiency (9).

The optimal sequence of PCV therapy within the HNSCC treatment paradigm remains under investigation, however emerging evidence suggests a benefit in the adjuvant setting. Following surgery, chemotherapy or radiotherapy, the tumor burden becomes significantly reduced, possibly lessening the immunosuppressive TME. The PCV TG4050 discussed previously was evaluated in this context, as the patients in arm A of this trial received TG4050 after the completion of primary treatment, and experienced no relapse after 2 years (63). Further studies with larger number of patients in this setting are needed to confirm this hypothesis.

## 7 Conclusion

Therapeutic vaccines aim to complement host immunity by identifying tumor-specific antigens and generate a more robust and lasting immune response. Although these vaccines have demonstrated the ability to induce antigen-specific immune responses, clinical activity has been limited. As HNSCC tumors evolve, so do their neoantigens, making general targetable mutations less effective across diverse patient populations. Among therapies under investigation, viral vector-based vaccines like TG4050, or DNA-based vaccines such as VB10.NEO are emerging candidates to overcome key challenges currently present in the management of HNSCC. Combining them with ICI can enhance immune activation through stimulation of both innate and adaptive immune responses. Future clinical trials should evaluate perioperative vaccine strategies in combination with ICIs to determine whether earlier administration can boost immune responses and improve outcomes. Understanding the gaps between robust immune activation and durable clinical activity is essential for advancing therapeutic cancer vaccination as a viable treatment modality for patients with historically poor outcomes.
